# Social deprivation affects cooperative predator inspection in a cichlid fish

**DOI:** 10.1098/rsos.140451

**Published:** 2015-03-25

**Authors:** Saskia Hesse, Jaime M. Anaya-Rojas, Joachim G. Frommen, Timo Thünken

**Affiliations:** 1 Institute for Evolutionary Biology and Ecology, University of Bonn, An der Immenburg 1, Bonn 53121, Germany; 2 Department of Fish Ecology and Evolution, Evolution and Biogeochemistry, Seestraße 79, Kastanienbaum 6047, Switzerland; 3 Department of Aquatic Ecology, Eawag Centre of Ecology, Evolution and Biogeochemistry, Seestraße 79, Kastanienbaum 6047, Switzerland; 4 Department of Behavioural Ecology, Institute for Ecology and Evolution, University of Bern, Wohlenstr. 50a, Hinterkappelen 3032, Switzerland

**Keywords:** cooperation, predator inspection, social isolation, cichlids, social environment

## Abstract

The social environment individuals are exposed to during ontogeny shapes social skills and social competence in group-living animals. Consequently, social deprivation has serious effects on behaviour and development in animals but little is known about its impact on cooperation. In this study, we examined the effect of social environment on cooperative predator inspection. Predator inspection behaviour is a complex behaviour, which is present in a variety of shoaling fish species. Often, two fish leave the safety of the group and inspect a potentially dangerous predator in order to gather information about the current predation risk. As predator inspection is highly risky, it is prone to conflicts and cheating. However, cooperation among individuals may reduce the individual predation risk. We investigated this complex social behaviour in juveniles of the cichlid fish *Pelvicachromis taeniatus* that were reared in two different social environments throughout development. Fish reared in a group inspected more often than isolation-reared fish and were more likely to cooperate, i.e. they conducted conjoint inspection of a predator. By contrast, isolation-reared fish were more likely to perform a single inspection without a companion. These results suggest an impairment of cooperative behaviour in isolation-reared fish most probably due to lack of social experience and resulting in lowered social skills needed in coordinated behaviour.

## 2. Introduction

The phenomenon of cooperation has puzzled natural scientists since Darwin's times (e.g. [1–4]). In the last 50 years considerable progress has been made concerning the understanding of the evolution of cooperation (e.g. [2,5,6]). Milestones were Hamilton's inclusive fitness theory, explaining cooperation among relatives and the ideas of reciprocity or mutualism mechanisms facilitating cooperation between unrelated individuals (for reviews see [2,7]). However, the developmental requirements necessary for the emergence of cooperative behaviour are less well studied.

The social environment that an individual is exposed to during its development has been suggested to crucially affect individual performance and thus may also contribute to the development of cooperative behaviour [8–11]. For example, in mammals, individuals reared in isolation or a less complex social environment (e.g. peer or maternal deprivation) showed an increase in stress hormone levels [12], atypical aggressive behaviour [13,14], increased anxiety in novel situations [15], socially incompetent or stereotypical behaviour [16,17] and altered brain development [17,18]. In a colonial bird (zebra finch, *Taeniopygia guttata*), the removal of adult males during early ontogeny had an impact on mating behaviour [19]. In fishes, the social environment affects a variety of behaviours, for example anti-predator behaviour, foraging and mate choice (for a review see [20]), sexual and filial imprinting (for a review see [21]) as well as aggression levels [10]. Furthermore, aggression, retention of memories and hormone levels are affected by short-term social isolation ([22–25]; for a detailed review see [26]). Arnold & Taborsky [27] report that juveniles of the cooperative breeding cichlid *Neolamprologus pulcher* raised in a more complex social environment—i.e. with adults (parents and/or brood care helpers)—showed more appropriate aggressive behaviour compared with juveniles reared in a less complex social environment (i.e. without adults), suggesting that the complexity of the social environment affects social competence and social skills (see also [11]). However, relatively little is known about the impact of social environment on more complex behaviour like cooperation.

Predator inspection is widespread in fishes (for example [28–31]). Inspecting fish gather information about the current predation risk and the information is transferred to other group members [28,32]. Acquiring information on a predatory threat is important in order to assess the immediate risk, e.g. hunger status of the predator [33]. Often, fish do not inspect alone but cooperate to dilute the risk and confuse the predator [29,34]. A companion—if close enough—shares the risk with the leading fish [34]. Therefore, predator inspection behaviour offers an excellent opportunity to study the impact of social environment during ontogeny on cooperative behaviour in a functional context. Cooperative predator inspection is a complex social behaviour that requires fine-tuned communication between inspecting individuals; it confronts them with the decision whether to take risks alone or cooperate with another individual and share them.

In this study, we aimed to investigate the effects of social deprivation on cooperation in juvenile *Pelvicachromis taeniatus.* Juvenile *P. taeniatus* live in shoals and are therefore an ideal study species to investigate anti-predator behaviour. We have previously demonstrated that social isolation negatively affected growth and behaviour in juvenile *P. taeniatus* [35]; thus we expected isolation-reared fish to show impaired anti-predator behaviour and be less willing to cooperate during predator inspection visits due to a lack of social competence.

## 3. Material and methods

### 3.1 Study species

*Pelvicachromis taeniatus* is a small, cave-breeding, socially monogamous cichlid from Western Africa with mutual mate choice and biparental brood care [36,37]. After several weeks of parental care, juveniles live in shoals until they reach sexual maturity [38]. Long-term isolation negatively affects growth, with fish reared in isolation being on average smaller than fish reared in a group [35]. Furthermore, social behaviour in juveniles reared in isolation is seriously impaired [35]. Juveniles reared in isolation are not able to recognize their relatives, indicating that juvenile kin recognition in this species most likely depends on familial imprinting [39].

### 3.2 Predators

We used five snakeheads (*Parachanna obscura*) as predators. Snakeheads are an established fish predator model for the study of anti-predator behaviour (e.g. [40,41]). *Parachanna obscura* is a sit-and-wait predator inhabiting the same natural habitat as *P. taeniatus* [42]. Five individuals (mean total length=13.25±s.d. 1.37 cm) were obtained from a commercial fish trader (PantaRhei Aquaristik) and housed individually in tanks (45×40×30 cm). Housing tanks were equipped with an internal filter, gravel, plants (java moss (*Taxiphyllum barbieri*); java fern (*Microsorum pteropus*)) and rocks to provide shelter. The water temperature was kept at 23±1°C; a light regime of 12 L : 12 D was used. Snakeheads were fed every 3 days with dead *P. taeniatus*.

### 3.3 Breeding of experimental fish

All experimental fish were bred under standardized conditions between April and October 2011 at the laboratory of the Institute of Evolutionary Biology and Ecology, University of Bonn, Germany. Breeding pairs of *P. taeniatus* (F_1_-generation of wild-caught fish) were assembled and each of them was introduced into a breeding tank (length×width×height: 45 cm×40 cm×30 cm) equipped with a standardized breeding cave, an aquarium heater, an internal filter, gravel and java moss (2.5 g). The water temperature was kept at 24±1°C and the experimental subjects were held under a light regime of 12 L : 12 D. They were fed daily with a mixture of defrosted *Chironomus* larvae, *Artemia* and black mosquito larvae. Approximately 30% of the water was changed weekly to increase spawning probability. Breeding caves were checked for eggs daily; eggs were transferred to a small plastic tank (16×9×10 cm) filled with tap water and equipped with an airstone for oxygen supply. Approximately two-thirds of the water was changed daily.

### 3.4 Rearing conditions of experimental fish

Eighteen breeding pairs spawned. Eggs were removed from the parents and raised artificially in small tanks (30×20×20 cm). At an age of 14±1 days fry were assigned to the treatment groups. For the group treatment, juveniles were kept in full-sibling groups of 10–15 fish (each family contributed two sibling groups). Each sibling group was housed in a tank (45×40×30 cm) equipped with sand, java moss and an internal filter. For the isolation treatment, up to six fish of each family were reared individually in tanks (30×20×20 cm) equipped with sand, java moss and an internal filter. All tanks were surrounded by opaque plastic sheets to prevent visual contact between inhabitants of different tanks. Free-swimming fry were fed with living *Artemia* nauplii provided in a highly concentrated suspension. Juvenile fish were fed daily ad libitum with a mixture of defrosted *Chironimus* larvae, *Artemia* and black mosquito larvae.

### 3.5 Experimental set-up

**Figure 1.**
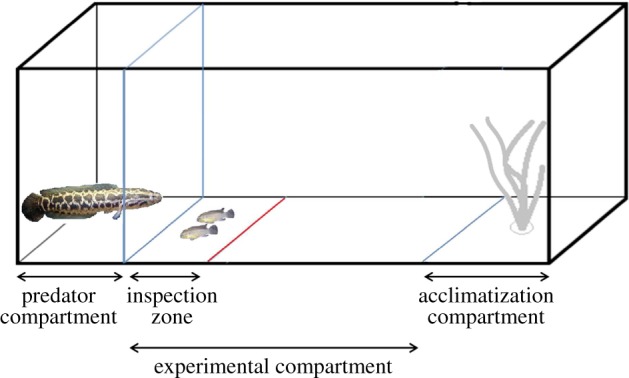
Experimental set-up viewed from the side. The experimental tank (70× 35×35 cm) was divided into four compartments: predator compartment (15 cm), and experimental compartment (38.5 cm) containing the inspection zone (22 cm) and an acclimatization compartment (16.5 cm). The predator compartment was separated from the rest of the tank by a transparent perforated plastic sheet. The acclimatization compartment containing a plastic plant as refuge was separated by a removable opaque plastic sheet. Figure is not true to scale.

The experimental tank (70×35×35 cm, water level 12 cm) was divided into three compartments: a predator compartment (15 cm), an experimental compartment (38.5 cm) and an acclimatization compartment containing a plastic plant as refuge (16.5 cm; figure 1; cf. [43]). The acclimatization compartment was separated from the rest of the tank by a removable opaque plastic partition to ensure an undisturbed acclimatization period. A transparent perforated plastic partition (permitting visual as well as olfactory contact between prey and predator) separated the predator compartment from the experimental compartment. The experimental compartment contained an inspection zone (22 cm) located directly in front of the predator compartment. The size of the inspection zone was based on the size of the predators (13.25±1.37 cm) and the highest predation risk relative to distance found in literature based on fast start performance of teleost fish [44,45] and an experiment on risk allocation [34]. Thus, predation risk was highest in this area and individuals should greatly benefit from cooperation. Furthermore, pretests showed that fish within the inspection zone clearly showed inspection behaviour, i.e. they purposefully approached the predator, stopped near the predator and then departed again slowly [28]. Behaviour in the inspection zone was therefore clearly different from normal shoaling behaviour. All compartments as well as the inspection zone were marked on the ground by black lines.

To avoid interactions of test fish with their reflections, the experimental tank was covered with grey plastic sheets on the inner sides. Additionally, the experimental tank was surrounded with white Styrofoam to minimize disturbance. The tank was filled with aged substrate-treated water (23±1°C; see [46] for details). After each experiment it was cleaned and rinsed with hot water and refilled with aged substrate-treated water.

Behaviour of test fish was recorded by a webcam (Logitech Webcam, Pro 9000) attached to a wooden frame 70 cm above the centre of the tank. The experimental tank was illuminated from above by a fluorescent tube (Osram Lumilux L58W).

### 3.6 Experimental procedure

To investigate juvenile cooperative predator inspection, a pair of immature, unfamiliar fish that were either reared in a group or reared in isolation was tested. Only fish from the same social rearing environment were tested together, i.e. isolation fish were only paired with isolation fish and group-reared fish were only paired with group-reared fish. Test fish were carefully netted and each was placed in a small plastic tank (17×10×10 cm, water level 5 cm). Characteristic fin patterns (dots) were recorded to recognize individual test fish. Test fish were immature and could not be sexed definitively. Each fish was only tested once.

A snakehead was carefully netted and introduced into the predator compartment. No predator was used more than twice each day. Then, test fish were transferred to the experimental tank by gently and simultaneously pouring them from the plastic tanks into the acclimatization compartment. They were allowed to acclimatize for 45 min. Subsequently, the opaque partition separating the acclimatization compartment from the experimental compartment was lifted using a pulley system. Since our experimental fish were predator naive individuals, we used a conspecific alarm cue (1 ml) that was added at the centre of the tank just before the trial started using a pipette to trigger a stronger anti-predator response, increase the vigilance status of the prey and to stimulate the predator [47,48]. Alarm cue extraction followed the protocol established by Meuthen *et al.* [49]. Each trial was recorded for 45 min. After the trial, the standard body length (SL) of the test fish was measured. There was no significant size difference between the dyads of the two different treatment groups (pairs of test fish reared in a group versus pairs of test fish reared in isolation; linear mixed effect model (LME): LRT, *N*_isolation_=15, *N*_group_=45, *χ*^2^=0.999, *p*=0.3174; size difference_isolation_=0.456 cm±s.d. 0.333, size difference_group_=0.407 cm±s.d. 0.358).

### 3.7 Data acquisition

Analyses of behaviours from videos were conducted blindly with regard to treatment (i.e. social environment). After one fish entered the inspection zone, the trial started and the consecutive 400 s were analysed. Snapshots were taken of each video every 5 s, i.e. 80 snapshots per trial. We chose this time frame since pilot trials revealed that test fish became habituated to the presence of predators after a while (S.H., personal observation). If both focal fish had not entered the inspection zone within 45 min, trials were excluded from analysis (*N*=5). We distinguish between two different types of inspection behaviour: (i) conjoint inspections or (ii) solitary inspections. In a conjoint inspection both fish entered the inspection zone either simultaneously or time-delayed. A conjoint inspection started when both fish had entered the inspection zone and ended as soon as one fish left the inspection zone, i.e. abandoned his companion. A solitary inspection was defined as only one fish entering the inspection zone or a fish being abandoned by his companion fish (thus remaining alone in the inspection zone); a single inspection event ended either when the inspector left the inspection zone or when the other fish entered the inspection zone (i.e. time-delayed conjoint inspection). We recorded the time (i.e. number of 5-s snapshots) test fish spent inspecting the predator either conjoint or solitary.

Furthermore, every frame was used to measure the distance of fish one to fish two in the software ImageJ v. 1.45 s. Median values of each video were used to analyse the data.

### 3.8 Data analysis

Statistics were performed with the R v. 2.9.1 statistical software package. Binomial and counting data were analysed using a generalized mixed effect model (GLMM; no overdispersion was detected). Furthermore, linear mixed effect models (LMEs) were used when original data were normally distributed according to Kolmogorov–Smirnov–Lilliefors tests and showed homogeneous variances according to Bartlett tests. Original data that failed normal distribution were log transformed. Reported *p*-values of models refer to the increase in deviance when the respective variable was removed. Tests of statistical significance were based on likelihood ratio tests (LRT), which follow a *χ*^2^-distribution. These routines use maximum-likelihood parameter estimation. Non-significant factors were removed from the models. The *p*-values are two-tailed throughout.

In total, 60 valid trials were performed (isolation treatment: *N*=15 (unfamiliar kin: *N*=8, unfamiliar non-kin: *N*=7); group treatment: *N*=45 (unfamiliar kin: *N*=21, unfamiliar non-kin: *N*=24)). To examine whether group- and isolation-reared fish differ in probability to perform a conjoint inspection within an experimental trial respectively, we conducted a GLMM with binominal distribution with conjoint inspection (occurrence within a trial yes/no) as dependent variable, social environment, size difference between test fish and genetic relatedness (in order to control for variation caused by different relatedness within experimental pairs, i.e. unfamiliar full siblings and unfamiliar non-siblings; genetic relatedness was kept in the model) as explanatory variables and family combination as random factor. The same GLMM was run with solitary inspection (yes/no) as dependent variable. To compare differences between the total number of inspections (single and conjoint inspections taken together) of isolation and group fish, a GLMM with Poisson distribution was run; total number of inspections was the dependent variable, social environment, size difference between test fish and genetic relatedness were the explanatory variables, and family combination was added as random factor. Additionally, we used a LME to investigate the effect of social rearing condition on time spent in cooperative inspections. Time spent in cooperation (time (s) inferred by the 5-s snapshots) was the dependent variable, and social environment, size difference between test fish and kinship were the explanatory variables; family combination was introduced as random factor. Furthermore, we analysed the mean distance between test fish for each trial using a LME with mean distance between test fish (log transformed) as dependent variable, social environment, size difference between test fish and kinship as explanatory variables, and family combination as random factor.

## 4. Results

**Figure 2.**
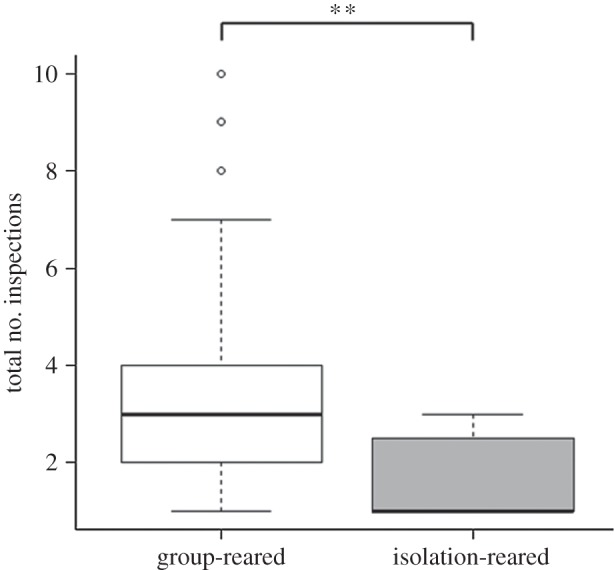
Average number of inspections per trial (solitary and cooperative inspections combined) of the two treatment groups. Median, first and third quartile and whiskers are shown. **p<0.01.

**Figure 3.**
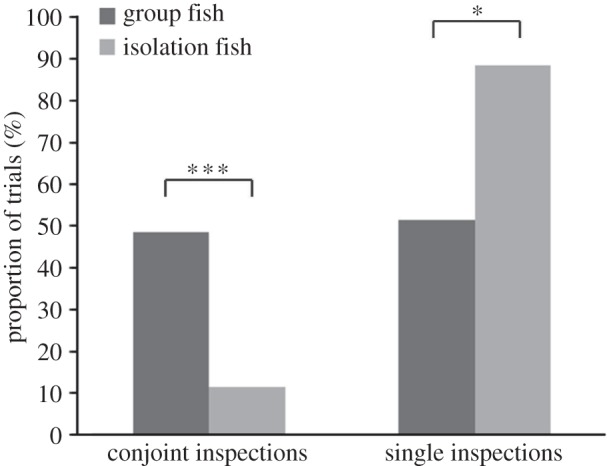
Percentage of test fish performing a conjoint and solitary inspection, respectively, relative to the total number of inspections depending on rearing environment (reared in isolation (light bars)/reared in a group (dark bars)). ***p<0.001; *p<0.05.

**Figure 4.**
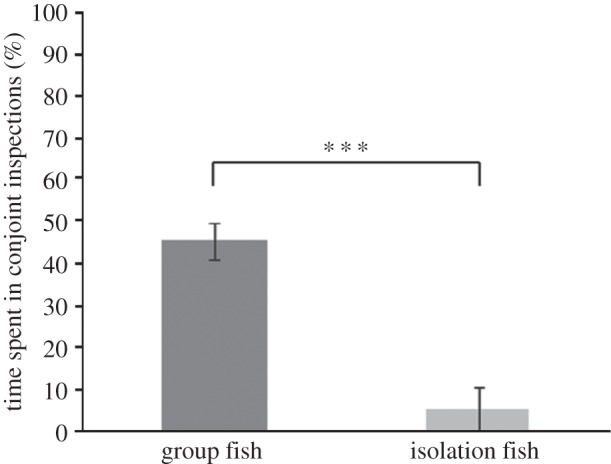
Mean time (%)±s.e. that test fish of the different treatment groups spent in conjoint inspections during the trial. ***p<0.001.

**Figure 5.**
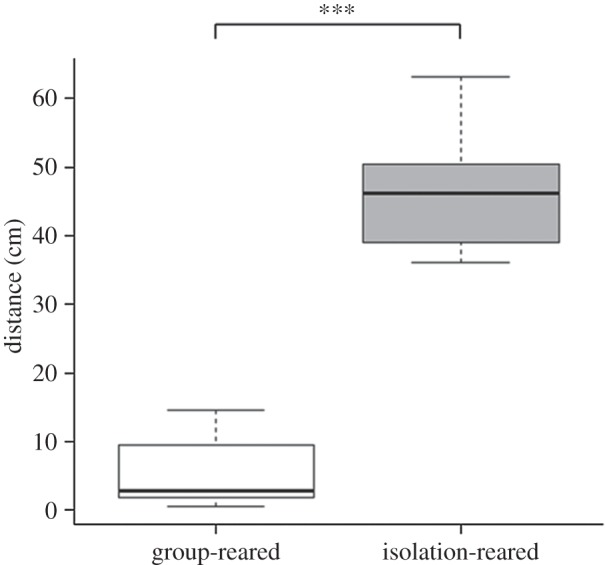
Distance (cm) between test fish of the different treatment groups. Median, first and third quartile and whiskers are shown. ***p<0.001.

Test fish reared in a group performed significantly more total inspections than isolation-reared fish (table 1 and figure 2). Size difference had no significant effect on total number of inspections. Test fish reared in a group were significantly more likely to perform a conjoint inspection than test fish reared in isolation (table 2 and figure 3). Size difference (table 2) had no significant effect on the occurrence of conjoint inspections. Group-reared fish also spent more time in conjoint inspections than did isolation fish (table 3 and figure 4). Size difference had no significant effect on the time test fish spent in conjoint inspections (table 3). By contrast, isolation fish were significantly more likely to perform a single inspection than group-reared fish (table 4). Size difference had no significant effect on the occurrence of single inspections (table 4). Test fish reared in a group also stayed on average closer together than isolation-reared fish (table 5 and figure 5). Size difference did not significantly influence distances between test fish (table 5).

**Table 1.** Results of a generalized mixed effect model (GLMM) analysing total number of inspections in relation to social environment (group-reared, *N*=45 and isolation-reared, *N*=15) and size difference between test fish. Family combination was included as random factor. Significant effects (*p*<0.05) are in bold font.

step	simplification	*χ*^2^	*p*-value	d.f.
maximum model				
1	size difference	0.002	0.964	1
2	social environment	12.719	<**0.001**	1

**Table 2.** Results of a generalized mixed effect model (GLMM) analysing occurrence of conjoint inspections (yes/no) in relation to social environment (group-reared, *N*=45 and isolation-reared, *N*=15) and size difference between test fish. Family combination was included as random factor. Significant effects (*p*<0.05) are in bold font.

step	simplification	*χ*^2^	*p*-value	d.f.
maximum model				
1	size difference	0.915	0.339	1
2	social environment	31.364	<**0.001**	1

**Table 3.** Results of a linear mixed effect model (LME) analysing time spent in conjoint inspection in relation to social environment (group-reared, *N*=45 and isolation-reared, *N*=15) and size difference between test fish. Family combination was included as random factor. Significant effects (*p*<0.05) are in bold font.

step	simplification	*χ*^2^	*p*-value	d.f.
maximum model				
1	size difference	1.643	0.199	1
2	social environment	24.641	<**0.001**	1

**Table 4.** Results of a generalized mixed effect model (GLMM) analysing occurrence of single inspections (yes/no) in relation to social environment (group-reared, *N*=45 and isolation-reared, *N*=15) and size difference between test fish. Family combination was included as random factor. Significant effects (*p*<0.05) are in bold font.

step	simplification	*χ*^2^	*p*-value	d.f.
maximum model				
1	size difference	0.031	0.861	1
2	social environment	6.277	**0.012**	1

**Table 5.** Results of a linear mixed effect model (LME) analysing the distance between test fish dependent on social environment (group-reared, *N*=45 and isolation-reared, *N*=15). Family combination was included as random factor. Significant effects (*p*<0.05) are in bold font.

step	simplification	*χ*^2^	*p*-value	d.f.
maximum model				
1	size difference	1.097	0.295	1
2	social environment	29.138	<**0.001**	1

## 5. Discussion

In this study, social rearing environment had profound effects on predator inspection behaviour in juvenile *P. taeniatus*. Test fish reared in a group performed significantly more predator inspections in total compared with isolation-reared fish and were significantly more likely to enter the inspection zone together, and thus perform a conjoint inspection. They also spent more time in conjoint inspections than isolation-reared fish did. A conjoint inspection indicates cooperation between inspecting individuals [29]. Conjoint inspection is beneficial because a companion fish dilutes the risk for the inspecting individual [34]. By contrast, isolation-reared test fish were more likely to perform single inspections compared to group-reared test fish, indicating less willingness or capability to cooperate. Thus, the social environment experienced during ontogeny is essential for the development of cooperative behaviour. Social behaviour is shaped by social interactions and the lack of conspecifics during this sensitive developmental period has serious consequences for social skills in juvenile *P. taeniatus*.

Cooperative predator inspection is a complex social behaviour that requires certain levels of social competence and cognitive abilities (e.g. [11]). Cooperative predator inspection among unrelated individuals often depends on reciprocity [29]. Mutual cooperation relies on communication and the ability to assess the intentions of companions. Social competence can be defined as an individual's ability to optimize and adjust its behaviour in a social context depending on the circumstances [50]. It is strongly influenced by social experience, as individuals may learn by the response of others to their behaviour or by observing interactions between conspecifics (eavesdropping) [20]. Previous social interactions can impact subsequent behaviour. For example, various studies showed that previous ‘winners’ of an aggressive encounter with a conspecific are more likely to win a subsequent fight with another individual. Consequently, ‘losers’ are more likely to lose their next aggressive encounter [51]. In addition, cooperative behaviour may also be affected by previous experience. This could for example be shown in Norway rats (*Rattus norvegicus*). Individuals that had received help before in a food acquisition task were more likely to help others as well when given the opportunity (generalized reciprocity) [52]. Thus, there are numerous ways in which previous experience may shape social behaviour. Isolation fish deprived of the company of other conspecifics had no chance to receive any social feedback or observe interactions between other fish. Therefore, reduced communicative abilities probably lead to single inspections and failure to carry out conjoint inspections successfully. Reduced communicative abilities are a consequence of social deprivation in many mammalian species. In rhesus monkeys (*Macaca mulatta*), socially deprived individuals reacted inappropriately in certain social situations, for example with aggressive behaviour in non-threatening situations [16], or failed to reconcile after aggressive encounters [53]. Rodents, especially mice (*Mus musculus*) and rats, are well-studied regarding the effects of social deprivation (mother–infant as well as peer deprivation) on social competence and behaviour. Effects of deprivation include reduced competence in agonistic encounters (e.g. to establish a territory, display and maintain social status, establish a hierarchy, etc. [17,54]) and marked differences in anxiety-like behaviour (for a review see [17]). In fishes, social learning has been investigated in numerous contexts, for example, foraging, anti-predator behaviour and mate choice (for a review see [20]). These aspects of social behaviour are influenced by interactions with and/or observation of other individuals. The complexity of a social rearing environment also affects behaviour and social competence [11]. Thus, our results suggest that social deprivation seriously impairs social skills in juvenile *P. taeniatus*, leading to reduced social competence and consequently lowered cooperative behaviour. Studies on social competence and skills suggested that the quality of a social environment has profound effects on social competence (e.g. [11]), which is most likely a precondition for more complex social interactions, e.g. cooperation. Our results further elucidate the profound impact social deprivation has on the ontogeny of social competence and deepen our understanding on how the presence of conspecifics shapes social skills.

We have previously demonstrated that isolation negatively affects behaviour in juvenile *P. taeniatus*; fish reared in isolation were more aggressive and less likely to shoal [35]. Aggression negatively affects vigilance and dense shoals are a major aspect of anti-predator behaviour in fish [55]. This could be shown for example in guppies (*P. reticulata*) where alarm cues induce an increase in shoal cohesion [55]. Increased shoal cohesion is likely to facilitate benefits gained by shoaling behaviour such as dilution and confusion effects. Lack of cooperative predator inspection in this study further reinforces that social deprivation impairs social competence. In this study, group-reared fish were also generally closer together than isolation-reared fish. Thus, both active anti-predator behaviour (predator inspection) and more passive anti-predator behaviour (shoaling) are seriously impaired in isolation-reared fish.

In summary, our study demonstrated that social deprivation crucially affects predator inspection in juvenile *P. taeniatus*. Conjoint predator inspections, which rely on cooperation between individuals and thus social communication, were significantly less likely to be performed by isolation-reared test fish. Therefore, our study highlights the importance of the social environment during ontogeny for the development of social behaviour and cooperation.

## Supplementary Material

data is attached as ESM

## Data accessibility

The dataset supporting this article is attached as electronic supplementary material.
